# Ultrastructural pathology of nephropathies with organized deposits: a case series

**DOI:** 10.1186/1757-1626-1-184

**Published:** 2008-09-25

**Authors:** Fabio Fabbian, Nevio Stabellini, Adriana Galdi, Sergio Sartori, Arrigo Aleotti, Luigi Catizone

**Affiliations:** 1 Renal Unit, St. Anna Hospital, Corso Giovecca 203, 44100 Ferrara, Italy; 2 Internal Medicine, St. Anna Hospital, Corso Giovecca 203, 44100 Ferrara, Italy; 3 Electron Microscopy Service, University of Ferrara, Via Savonarola 9, 44100 Ferrara, Italy

## Abstract

Renal organized or structured deposits are much less frequent than those with usual type immunocomplex deposits and are encountered in a wide variety of primary and systemic disorders. It has been suggested that immunoglobulins (Igs) are responsible for organized deposits. We report 5 cases who have been diagnosed and treated in our hospital. Patients were aged 52 to 72 years, three of them were males and had variable degree of renal function, from normal serum creatinine to uraemia. Proteinuria was detected in all patients while monoclonal component was present only in the serum of one subject. Ultrastructural analysis of renal specimens revealed organized deposits. Diagnoses that were made are the following: membranoproliferative glomerulonephritis with finger print, immunotactoid glomerulopathy, membranoproliferative glomerulonephritis with arched deposits, primary amyloidosis and light chain deposition disease. In systemic disorders ultrastructural pathology could be particularly valuable for correct deposits classification, precise localization and pattern of deposition of Igs.

## Introduction

Differential diagnosis of renal organized or structured deposits is a matter of debate [1]. They are much less frequent than those with usual type immunocomplex deposits and are encountered in a wide variety of primary and systemic disorders [2]. Organized deposits characterization depends on and appears to be related to specific diseases [1,3-6]. In the last years there has been an improvement in understanding of these rare finding with benefits in clinical management of systemic disorders. Electron microscopy (EM) analysis has been crucial, in fact by light microscopy, these entities may mimic different patterns. Aim of our study was to report our experience about morphological characterization of organized deposits.

## Case presentation

### Case 1

**Figure 1:**
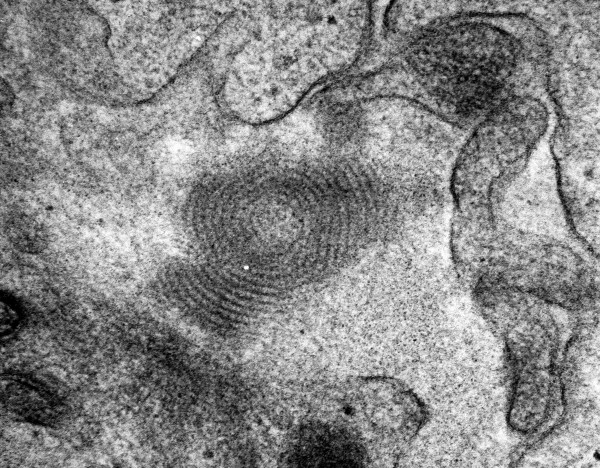
Fingerprint-like intramembranous deposit (magnification ×60000).

A 64-year-old Caucasian man was referred to our unit because of mild hypertension and peripheral oedema. Renal function was normal but he had proteinuria (3.5 g/24 h). His serum albumin was 25 g/L associated with high immunoglobulins level. Serum C3 and C4 were normal, he had abnormal liver function, markers for hepatitis C virus (HCV) infection were negative whilst HBsAg was positive. Cryoglobulins were not detected. An abdominal ultrasound showed increased in liver and spleen volume. Liver and renal biopsies were performed and revealed hepatitis and membrano-proliferative glomerulonephritis associated with "finger-print" deposits (Figure 1).

### Case 2

**Figure 2:**
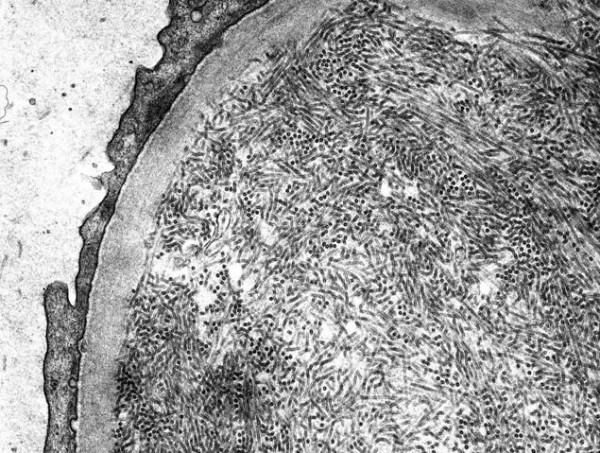
Deposit characterized by hollow structure (magnification ×15000).

A 72-year-old Caucasian man was hospitalized because of acute renal failure (serum creatinine 4.4 mg/dl). His clinical history included arthralgias, hypertension, necrotizing leucocytoclastic vasculitis and in his serum a monoclonal component was identified (IgAλ 9.8%). Bone biopsy had atypical plasma cells (8%). Proteinuria was 1 g/24 h associated with haematuria, but Bence-Jones proteinuria was negative. Renal biopsy was carried out that diagnosed an immunotactoid glomerulopathy. EM showed subendothelial and mesangial deposits of structurated microtubules which diameter was 50–60 nm (Figure 2).

### Case 3

A 51-year-old Caucasian lady was hospitalized because of purpuric papules of the lower extremities. She complained of myalgias and arthralgias. Her renal function was normal but she had, haematuria and proteinuria (1.2 g/24 h). Laboratory work-up discovered that C_3 _was mildly while C_4 _was significantly reduced. HCV infection was not found and cryocrit was 0.5%. Bence-Jones proteinuria was negative. She underwent skin biopsy that showed leucytoclastic vasculitis and subsequently renal biopsy. Examination showed membrano-proliferative glomerulonephritis. Ultrastructural examination evidenced mesangial, subendothelial and subepithelial organized electron-dense deposits characterized by arched fibrils with a diameter of 24 nm. Cryoglobulinaemia type III was diagnosed.

### Case 4

**Figure 3:**
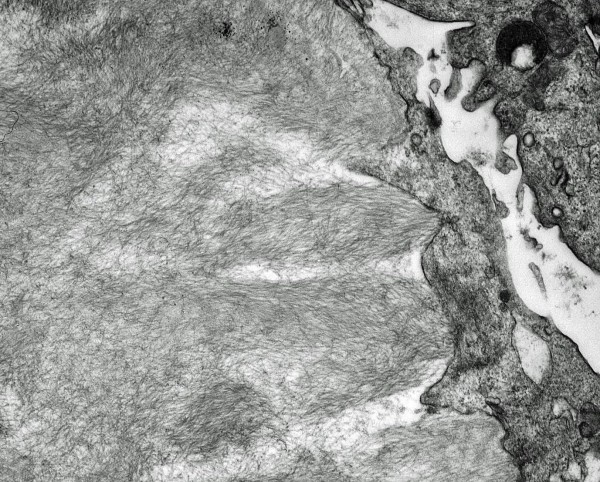
Randomly distributed, non-branching fibrils (magnification ×15000).

A 63-year-old Caucasian man was admitted because of nephrotic syndrome and renal failure (serum creatinine 1.6 mg/dl). Proteinuria was 8–9 g/24 h and Bence Jones proteinuria was positive. Renal biopsy showed massive amyloid deposition and fibrils infiltrating various renal compartments (Figure 3). Primary amyloidosis was diagnosed.

### Case 5

A 55-year-old woman was admitted because serum creatinine was 9.4 mg/dl, potassium was 6.8 mmol/l and was anaemic (haemoglobin 7.7 g/dl). Proteinuria was 1 g/24 h associated with haematuria. Immunofixation showed monoclonal kappa light chain in the urine. The patient underwent renal biopsy. Light microscopy examination showed smooth and continuous deposition of eosinophil material in the tubular basement membrane, mild thickening and stiffness of the glomerular basement membrane, and increase of the mesangial matrix. EM examination displayed coarse granular electron-dense deposits in the outer surface of the tubular basement membranes, nonfibrillar electron dense material along the glomerular basement membrane and in the mesangium. Bone marrow aspiration and bone biopsy were performed, and histologic examination of the specimens confirmed the diagnosis of monoclonal immunoglobulin deposition disease associated to kappa light chain multiple myeloma.

## Discussion

We report 5 cases of renal syndromes in which ultrastructural analysis of kidney specimens had a crucial role distinguishing between usual type immunocomplex deposits and organized ones and this information could be important for clinical management of patients.

Renal organized deposits (ROD) are usually due to immunoglobulins (Igs) or Igs-related material deposition or precipitation.

The generic term glomerular deposition disease (GDD) has been proposed by pathologists in order to identify nephropaties with ultrastructural ROD including glomerular diseases or paraproteinemic diseases or haemopoietic diseases [3,6]. In fact presence of ROD always suggests a systemic disease. ROD require by physicians to detect autoantibodies, paraproteins in the serum or in the urine and a bone marrow check in order to diagnose not only the renal disease, but also the haemopoietic diseases, responsible for Igs or Igs-related material production from plasma cells with monoclonal or polyclonal proliferation [6]. GDD has been classified in three entities according to the characteristics of glomerular deposition that could be fibrillar, monoclonal or polyclonal material. Monoclonality of the glomerular deposit is not determined by serum or urine monoclonal Igs or Igs subunits, but according to monoclonal deposition on the glomeruli on immunofluorescence of the subtypes of Ig or light chain, kappa or lambda. There are many cases of dissociation between monoclonality of the deposition and monoclonality in the serum [3]. EM recognizes three different types of ROD including crystals such as in myeloma cast nephropathy, fibrils such as in amyloidosis and fibrillary glomerulonephritis and microtubules such as in cryoglobulinaemia and immunotactoid glomerulonephritis [7]. A different category of diseases could be considered those characterized by non-organized electrodense granular deposits, mainly localized along basement membranes in most tissues such as in monoclonal immunoglobulin deposition disease (MIDD) [7].

In 1997 the Renal Immunopathology Study Group of the Italian Society of Nephrology published a survey of the Italian Registry of Renal Biopsies analyzing 13835 biopsies performed in native kidney over a period of 7 years (1987–1993). Incidence of dysgammaglobulinaemia associated nephritis was 2.4 per million of population (renal amyloidosis 0.9, essential mixed cryoglobulinaemia 0.9, multiple myeloma 0.3, light-chain disease 0.1) [8].

We conclude that, although expensive, ultrastructural analysis of deposits could be useful especially when systemic disease is suspected.

## Competing interests

The authors declare that they have no competing interests.

## Authors' contributions

FF, NS, AG, SS, AA, LC performed the clinical work and made the diagnosis, acquired, analyzed and interpreted the data. FF, NS and AG drafted the manuscript. SS performed the investigations. AA performed the electron microscopy work. All authors read and approved the final manuscript.

## Consent

The patients were informed about the article and hypothetical beneficial effects of its publication on clinical practice. Written informed consent was obtained from the patients for publication of this Case report and any accompanying images. A copy of the written consent is available for review by the Editor-in-Chief of this journal.
